# News and Coming Events

**Published:** 1908-08-29

**Authors:** 


### News and Coming Events.

The University of Madrid is making arrangements to celebrate, on October 18, 1906, the four hundredth anniversary of its foundation.

The Emperor William has agreed to the making of a grant of 100,000 marks (£5,000) from the contingency fund to the Robert Koch Institute for combating tuberculosis.

The Colchester Education Committee has decided to appoint a lady doctor as assistant medical officer of health, to carry out the duties connected with the medical inspection of school children.

Dr. W. H. Willcox, lecturer on public health, pathological chemistry and forensic medicine in St. Mary's Hospital Medical School, and physician to out-patients at that hospital, who has been for some time scientific analyst, has been appointed senior scientific analyst to the Home Office in succession to the late Sir Thomas Stevenson.

Dr. E. G. Newell, of Moville, co. Donegal, was recently presented with a testimonial consisting of a purse of 125 sovereigns and a silver cigarette-box bearing a suitable inscription by his many friends in Innishowen, as a mark of their appreciation of his long and valued services in the district.

The *Cologne Gaette* states that the question of the admission of Women to the German University study has been settled. Women who are subjects of the Empire will be admitted on the same footing as men, but women of other countries will require the permission of the Minister of Public Instruction for matriculation.

Dr. Cook, Medical Officer for the Tendring Rural District of Essex, expresses the opinion, in his latest report, that "the greatest misfortune that ever happened to the dairy business was the abolition of the milkmaid. She could have, and generally had, clean hands, but to get the hands of the present cowman, who does all the dirty work about the place, clean is an utter impossibility."

A special appeal for funds is made on behalf of the Invalid Children's Aid Association, which has recently been forced to greatly enlarge its sphere of charitable work by including the children of West Ham. In 1906 1,390 new cases were added to the list of the Association, and 1,400 in 1907, while up to the present time there have been 250 more cases this year than for the same period last year. Donations should be sent to the Secretary, Denison House, Vauxhall Bridge Road.

The First International Moral Education Congress will be held at the University of London on September 25 to 29, 1908. Further information may be obtained from the General Secretary, Gustav Spiller, 13 Buckingham Street, Strand, London.

The trustees of the will of the late Theodotus John Sumner are setting aside securities representing £20,000 for the purpose of assisting in the rebuilding of the Melbourne Hospital. These securities represent part of the moneys directed by the will of the late Mr. Sumner to be employed in charitable purposes. The trustees will retain the funds, together with accumulating income, until they are required for the objects named.

A report dealing with the relationship between the consumption of mussels and typhoid fever has been prepared by Dr. Duchan, assistant medical officer for Birmingham. He found that in 62 out of 74 cases of typhoid notified during four years the onset of the disease occurred during the height of the mussel season. Experiments showed that mussels readily absorbed the typhoid bacilli from polluted water. Twenty-five samples of raw mussels from fifteen markets were examined, and many bore evidence of having been exposed to pollution.

The death occurred on August 13, at the age of eighty-two, of Sir Charles Gage Brown, for many years medical adviser to the Colonial Office. He was born in Dorsetshire in 1826 and after studying at King's College Hospital he qualified M.R.C.S.Eng. in 1847 and M.D. (St. Andrews) in 1851. He subsequently became Member and Fellow of the Royal College of Physicians of Edinburgh and LL.D. Honoris Causâ of St. Andrews. From 1874 until 1897 he held the office of medical adviser to the Colonial Office and the Crown Agents for the Colonies, and on his retirement he was created K.C.M.G. in acknowledgment of his services.

According to the *Times* Pekin correspondent, China shows no relaxation in her anti-opium policy; but a formidable difficulty is the immense importation of morphia and hypodermic appliances. All the Powers except Japan have given their assent to the enforcement of the clauses of the American and British treaties of 1902 forbidding the importation of morphia except for medicinal purposes, and the assent of Japan is expected daily. An Imperial edict has recently been published, decreeing that Chinese who manufacture morphia or hypodermic appliances, or shopkeepers who sell morphia without a Customs permit, shall be banished to "a pestilential frontier of the Empire" and have their shops closed.

Lieut.-Colonel Mathias, R.A.M.C., has been appointed principal medical officer to the Egyptian Army.

"Anonyma" has given £1,000 towards the maintenance of a bed in the new wing of the Cardiff Infirmary.

Sir James Crichton-Browne is the President this year of the Sanitary Inspectors' Association Conference to be held at Liverpool, commencing September 7. On the morning of the 8th there will be a reception of delegates by the Lord Mayor.

The fourth International Electro-Therapeutic Congress will commence its session at Amsterdam on September 1. The congress this year will be held in the University Buildings at Amsterdam, which have been placed at its disposal. The President is Dr. J. K. A. Salomonson, Professor of Neuropathology.

A new development at the Post-Graduate College, West London Hospital, Hammersmith, is the establishment of a special course of six lectures on "The Medical Inspection of School-children." This course embraces points to be observed in the medical inspection of children and in the examination of their teeth, eyes, throat, nose, and skin. The course will begin on September 7 and will be completed in one week; the fee is £1 1s.

A vacation course, to be held during the first fortnight in September, has been arranged in connection with the North-East London Post-Graduate College. A syllabus, which may be obtained from the Dean, gives particulars of clinical demonstrations, classes of instruction in clinical methods, the bacteriology of daily practice, and clinical pathology, together with clinical lectures and lantern demonstrations. The fee for the course is one guinea.

The Local Government Board is issuing this week an important circular on the question of food inspection. The value of food imported into this country is over £200,000,000, and the circular will give instructions and advice to local authorities concerning more than half of this amount. The contents of the circular are the outcome of careful inquiries and investigations undertaken by the Board as a result of the "scare" of two years ago, and the Board believe they will have a most beneficial effect in securing the purity of the food of the people. Next week the Local Government Board will issue the expected circular dealing with the motor problem.

The Duchess of Sutherland on August 21 opened a bazaar, at Beaufort Castle, in aid of the Inverness-shire Sanatorium for Phthisis. The bazaar was held in a huge marquee in front of the castle, and there was a very distinguished assembly of stallholders and purchasers. The Duchess of Sutherland, in declaring the bazaar open, pointed out that phthisis was common in the Highlands, and that the remedies were largely pure air during the day and night, good food, and wholesome work These things, to a certain extent, had still to be taught in the Highlands, but much would be done by such sanatoria as had been established in Ross-shire by the munificence of Mrs. Stewart Mackenzie, of Seaforth, by the munificence and intelligence of the people of Inverness-shire, and especially by the organisation and perseverance of Miss Margaret Fraser. Such institutions would be of enormous educative value. She looked forward to the time when the people of the Highlands would refuse to live any life but a life that was wholly healthy.

The Rt. Hon. Winston Churchill, M.P., will open the new wing of the Jewish Hospital in Manchester on Tuesday, October 13.

Major Probyn, of the Royal Army Medical Corps, has been appointed sanitary specialist in South China. This is a new appointment.

It is announced in the *London Gazette* that the Army Council has decided to discontinue appointments to the Army Medical Reserve of Officers.

Owing to an outbreak of small-pox in Christiania, it has been decided to close the public schools until September 1. Up to August 25 there had been seventy-five cases and three deaths.

An appeal is being issued by a committee, headed by the Duke of Fife and the Archbishop of York, to present a national testimonial to Mr. Francis George Heath, who was the pioneer in the movement for preserving open spaces, woods, and forests, and who has for nearly forty years worked to secure that object, among the open spaces preserved to the public largely through his efforts being the Victoria Park Crown Lands, Epping Forest, and Burnham Beeches. Subscriptions will be welcomed by the hon. secretary and treasurer, Mr. Eugene de Rutzen, 17 Lawford Road, Kentish Town.

